# *Gurya* cutting and female genital fistulas in Niger: ten cases

**DOI:** 10.1007/s00192-017-3370-z

**Published:** 2017-06-19

**Authors:** Itengre Ouedraogo, Regina McConley, Christopher Payne, Alison Heller, L. Lewis Wall

**Affiliations:** 1 Danja Fistula Center, Danja, Niger; 2 Vista Urology and Pelvic Pain Partners, San Jose, CA USA; 3 Worldwide Fistula Fund, Chicago, IL USA; 4 School for Advanced Research, Santa Fe, NM USA; 5 Department of Anthropology, Washington University in St Louis, Campus Box 1114, St Louis, MO 63130-4899 USA

**Keywords:** Female genital cutting, Female genital mutilation, Genitourinary fistula, Hausa, Obstetric fistula, Vesicovaginal fistula

## Abstract

**Introduction and hypothesis:**

The objective was to determine the contribution of female genital cutting to genital fistula formation in Niger from the case records of a specialist fistula hospital.

**Methods:**

A retrospective review was undertaken of the records of 360 patients seen at the Danja Fistula Center, Danja, Niger, between March 2014 and September 2016. Pertinent clinical and socio-demographic data were abstracted from the cases identified.

**Results:**

A total of 10 fistulas resulting from *gurya* cutting was obtained: 9 cases of urethral loss and 1 rectovaginal fistula. In none of the cases was genital cutting performed for obstructed labor or as part of ritual coming-of-age ceremonies, but all cutting procedures were considered “therapeutic” within the local cultural context as treatment for dyspareunia, lack of interest in or unwillingness to engage in sexual intercourse, or female behavior that was deemed to be culturally inappropriate by the male spouse, parents, or in-laws. Clinical cure (fistula closed and the patient continent) was obtained in all 10 cases, although 3 women required more than one operation.

**Conclusions:**

*Gurya* cutting is an uncommon, but preventable, cause of genital fistulas in Niger. The socio-cultural context which gives rise to *gurya* cutting is explored in some detail.

## Introduction

In affluent countries such as the USA, UK or the Netherlands, genitourinary fistulas are rare. Fistula formation in affluent settings usually occurs as an unexpected complication of surgery (such as hysterectomy), radiation therapy, or malignant disease [1–3]. Obstetric fistulas from prolonged obstructed labor are extremely rare in resource-rich countries, yet such injuries cause most genitourinary fistulas in poor nations, where hundreds of thousands (if not several millions) of cases may still be found [4–6]. We report here on a less common, but completely preventable, type of genital fistula that occurs in resource-poor African countries as the result of a specific type of genital cutting locally called *gurya*. In such instances, genital fistulas are inadvertently created as the direct result of cutting the external genitalia, urethra, vagina or rectum. As with the post-hysterectomy fistula, this too is a form of surgical misadventure occurring in the setting of traditional therapeutic practices. We report a series of 10 such cases from the West African nation of Niger (all successfully managed) and discuss the complicated social and cultural circumstances in which such injuries arise.

## Materials and methods

The medical records of the Danja Fistula Center in Danja, Niger, were reviewed to uncover cases in which female genital cutting was the cause of genital fistulas. As there is no formal institutional review board at this institution, permission for the chart review was obtained from the medical director of the Danja Fistula Center. Because it involved only a review of medical records, the project was regarded as not requiring further monitoring. The charts were reviewed and pertinent clinical and socio-demographic data were extracted. Only 10 cases of fistula resulting from genital cutting procedures occurred out of a total of 360 cases seen in the period from March 2014 to September 2016. The other cases were all of obstetrical origin. These cases have been described elsewhere in detail [6] Nine of the 10 genital cutting cases involved a total loss of the urethra and the remaining case was a rectovaginal fistula from a cutting procedure. All 10 cases can be categorized as instances of *gurya*, a local form of traditional cutting therapy carried out for a variety of gynecological complaints.

Pre-operative evaluation was limited to history and physical examination. Urodynamic studies were not performed. Patients with a positive empty bladder stress test or a strong history of severe/total urinary incontinence were assumed to have an incompetent bladder neck and a sling was performed along with the neourethral reconstruction.

**Fig. 1:**
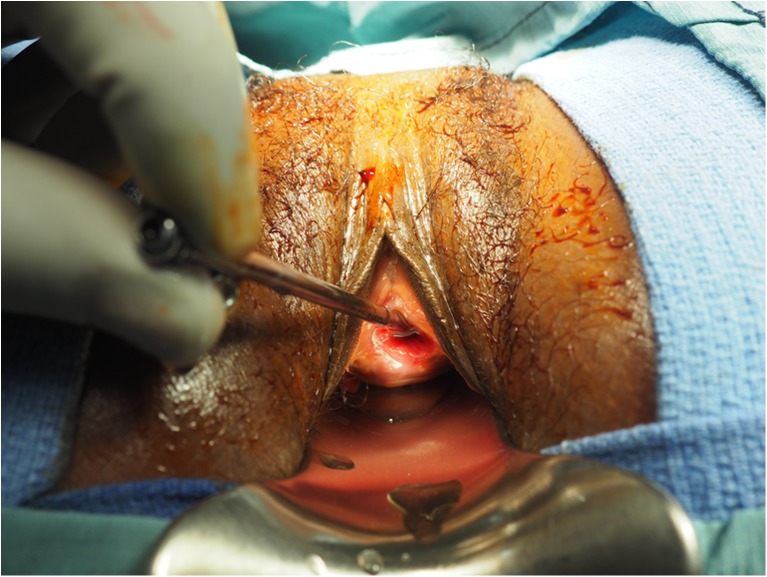
Urethra amputated by *gurya* cutting. Metal sound within the urethral stump at the bladder neck

**Fig. 2:**
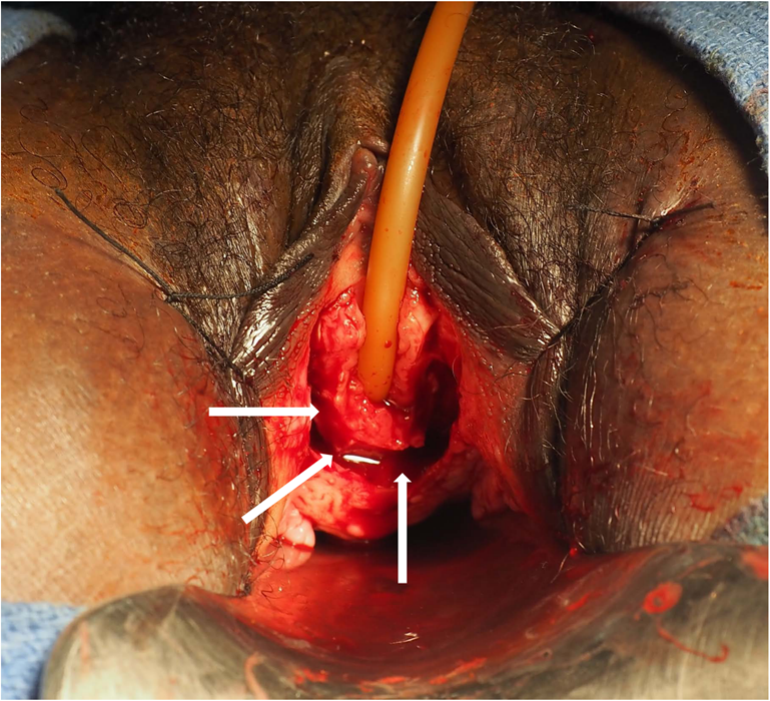
Dissection of flaps for the neourethra, indicating the extent of previous urethral loss. *Arrows* indicate the edge of the U-shaped incision

**Fig. 3:**
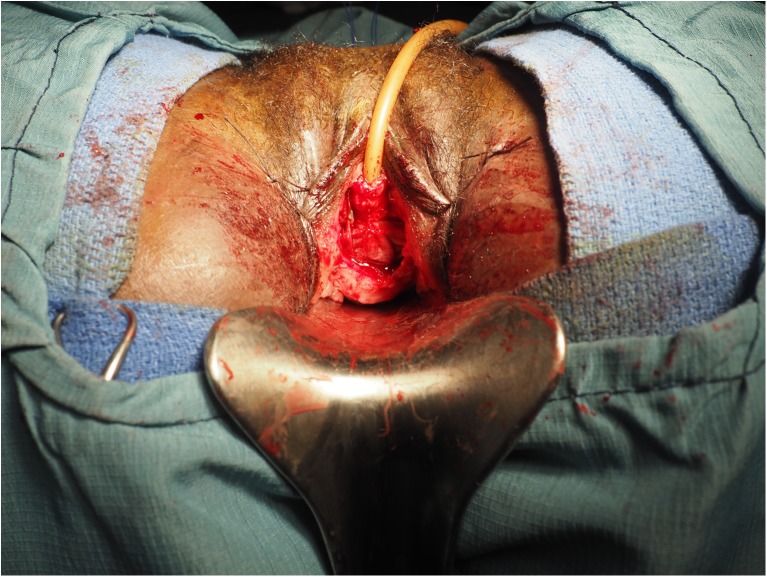
The flaps are closed to create the neourethra

The external genitalia were intact in all cases, but in the urethral injury cases, the urethra had been completely excised back to the bladder neck (Fig. 1). To create a neourethra, a U-shaped incision was made with the apex proximal to the existing urethral stump with the lateral arms of the “U” extending to the site of the former meatal opening (Fig. 2). The edges of the incision were mobilized both medially and laterally and the new urethra was then created by suturing the medial edges of the flaps together across the midline using interrupted 5–0 sutures of polyglactin 910 (Vicryl™; Ethicon, New Brunswick, NJ, USA), over a 14-French catheter, which served as a stent (Fig. 3).

For most cases in which a sling was included, a proximal dissection was made to expose the bladder neck and the dissection was extended along the periurethral fascia to enter the retropubic space, as has been described by McGuire and others [7]. A 2-cm by 8-cm strip of fascia lata was harvested through a single small lateral incision made 2 cm above the epicondyle of the knee. Sutures of 0-polypropylene (Prolene™; Ethicon) were placed at the two ends of the fascial strip and passed through the vaginal incision into the retropubic space using a tonsil clamp and out through the abdominal wall through a single midline suprapubic incision. The passage of the clamp through this space was continually guided with a fingertip. Passage of the sling was done before closure of the neourethra to avoid inadvertent injury to the delicate repair. After neourethral reconstruction, the sling was positioned at the level of the bladder neck and secured in place using sutures of 3–0 polyglactin 910 (Vicryl™; Ethicon). The sling was tied under very light tension to avoid creating an obstruction and the skin was closed under minimal tension to complete the repair. In two cases, a fibromuscular sling as described by Browning was used instead of a fascia lata sling, at the discretion of the operating surgeon [8]. In this technique, the lateral vaginal sidewall is opened, the ischiocavernosus muscle is mobilized bilaterally, the two ends of the muscle are brought together in the midline just under the (neo)urethra, and they are then sutured to one another to form the supportive sling [9].

## Results

Clinical characteristics of the patients and their surgical histories are given in Table 1 and the social characteristics are shown in Table 2. The ethnic background of these women is similar to that of the obstetric fistula patients seen at the Danja Fistula Center [6]. They were predominantly Hausa, with some Fulani and Tuareg patients. The obstetrical histories of these women were quite different from those of women who presented with fistulas resulting from obstructed labor. Six of the women with fistulas from genital cutting had never been pregnant, 3 had had only one pregnancy each, and 1 woman had had 6 pregnancies. In all 10 cases, the external genitalia were normal. None of these women had undergone type 1, 2, or 3 genital mutilation as defined by the World Health Organization (Table 3).

**Table 1:** Clinical characteristics of genital cutting cases

Number	Age (years)	Type of fistula	Operative treatment	Clinical outcome
1	22	Total urethral loss; intact bladder neck; severe vaginal scarring	Neourethra; discharged dry, but had recurrent lateral fistula at 6 months with partial loss of neourethra; repeat neourethra with fistula closure	Closed and dry 6 months after second operation
2	23	Total urethral loss with damaged bladder neck	Neourethra, failed; repeat neourethra with persistent incontinence; repeat neourethra with fascia lata sling	Closed and dry after third operation
3	31	Total urethral loss; intact bladder neck; mild scarring	Neourethra with Browning fibromuscular sling; wet owing to loss of neourethra 12 months later; repeat neourethra with Browning fibromuscular sling	Closed and dry 3 months after second operation
4	23	Total urethral loss, bladder neck intact	Neourethra with fascia lata sling	Closed and dry 3 months after operation
5	23	Total urethral loss; partial damage to bladder neck; mild scarring	Neourethra with anastomosis to bladder neck, fascia lata sling	Closed and dry 34 days post-operatively
6	12	2-cm mid-vaginal recto-vaginal fistula from cutting, moderate scarring	Rectovaginal fistula repair	Closed and continent
7	16	Total urethral loss; bladder neck damaged; mild scarring	Neourethra with fascia lata sling	Closed and dry 45 days post-operatively
8	15	Total urethral loss; mild scarring	Neourethra	Closed and dry 36 days post-operatively
9	19	Total urethral loss; bladder neck intact; mild scarring	Neourethra with fascia lata sling	Closed and dry 45 days post-operatively
10	18	Total urethral loss; bladder neck intact; mild scarring	Neourethra with Browning fibromuscular sling	Closed and dry 22 days post-operatively

**Table 2:** Social circumstances of genital cutting cases

Case	Age (years)	Age at marriage	Number of pregnancies	Age at cutting	Ethnicity	Marital status	Reason for cutting
1	22	16	1	20	Tuareg	Married	Unknown
2	23	14	1	Unknown	Fulani	Married	Unknown
3	31	Unknown	6	5, 30	Hausa	Married	Cut once in childhood for unknown reasons; developed pain and prolapse after her sixth pregnancy. Traditional healer told her the cutting was “incomplete” and needed to be redone, causing a fistula
4	23	14	0	18	Hausa	Separated	Husband was sexually abusive. She tried to escape him and her parents took her to a traditional healer to find a remedy for her sexual behavior
5	23	15	0	17.5	Hausa	Divorced	Husband was physically and sexually abusive. She tried to escape several times
6	12	12	0	12	Hausa	Separated	Husband was physically and sexually abusive. Was told she was “sick” and needed the operation so she would stay with her husband. Married 8 months before cutting took place
7	16	16	0	16	Hausa	Separated	Ran away from her husband because of nonconsensual, painful intercourse and was told she had *gurya*. Married 4 months before cutting
8	15	15	0	15	Hausa	Separated	Unknown
9	19	Unknown	1	Unknown	Fulani	Divorced	Ran away from husband because of nonconsensual intercourse. Stayed with her husband due to having a baby. Her co-wives convinced her husband to divorce her
10	18	16	0	16	Hausa	Divorced	Fled from her husband because of forced nonconsensual intercourse. Had the cutting and was given divorce papers. Reconciled with her husband, but he said the first procedure was “incomplete” and she had it done again, resulting in a fistula

**Table 3:** World Health Organization Classification of Female Genital Mutilation^a^

Type of cutting	Description
Type 1: clitoridectomy	Partial or total removal of the clitoris. In very rare cases, only the prepuce of the clitoris is removed
Type 2: excision	Partial or total removal of the clitoris and the labia minora, either with or without excision of the labia majora
Type 3: infibulation	Radical reduction and narrowing of the vaginal orifice by cutting and removing the labia minora and majora, often stitching the raw surfaces together, either with or without clitoridectomy
Type 4: other	All other harmful procedures performed on the female genitalia for nonmedical purposes, such as pricking, piercing, incising, scraping, or cauterizing the genital area

^a^Based on the World Health Organization Fact Sheet *Female genital mutilation*, updated February 2016 (http://www.who.int/mediacentre/factsheets/fs241/en/) accessed 16 October 2016

The outcome of surgery was good in all 10 cases. The 9 women with urethral loss all had successful neourethral reconstruction (combined with some form of sling operation in 7 cases) and all eventually regained continence. In 2 patients it took two operations, and in 1 patient it took three operations, for the women to become “closed and dry.” The rectovaginal fistula in the 12-year-old was successfully repaired. In this case, the vaginal tissues around the fistula were mobilized extensively and the fistula was closed transversely in a single layer using interrupted sutures of 3–0 polyglactin 910 (Vicryl™; Ethicon). The overlying vagina was then reapproximated with interrupted sutures of 2–0 polyglactin 910 (Vicryl™; Ethicon).

## Discussion

According to the World Health Organization, female genital mutilation “comprises all procedures that involve partial or total removal of the external female genitalia, or other injury to the female genital organs for nonmedical reasons” [10]. Most of these female genital cutting procedures are performed for ritual and prophylactic purposes—often integrated into initiation ceremonies for cohorts of young women and girls. Although local logics vary across cultural groups, common reasons for the practices include the alleged reduction of female desire (and thus, reducing hypersexuality and promiscuity); the “cleansing” of girls and women; and ritual circumcision (analogous in meaning to procedures performed on boys and men) [11]. The extent of the anatomical damage incurred through the procedures varies, and loosely correlates with the WHO’s classification of four types of cutting (Table 3). The cases presented here are all unusual manifestations of type 4 cutting, which are often less severely mutilating than the others, and differ from typical presentations of female genital mutilation in important ways. First, although female genital cutting is typically considered prophylactic and often practiced widely at the community level, the genital cuttings seen in this study were all performed therapeutically for individual cases of perceived gynecological or behavioral disorders. Indeed, these differing approaches to genital cutting are reflected in Mali’s 89% prevalence rate of female genital cutting among women aged 15 to 49 (mostly type 1 and type 2), whereas in Niger, the comparable rate is only 2% [12].

Despite the recognized risks of all forms of female genital cutting, which include sepsis, scarring, dyspareunia, infertility, psychological problems, hemorrhage, and sometimes death, within the understanding of traditional ethnomedical systems, these procedures are performed for what appear locally to be legitimate therapeutic purposes. Although there is no basis in biomedicine for the underlying assumptions that guide these practices, these systems of belief may nevertheless play a powerful role in directing the quest for therapy undertaken by patients and their families in parts of the world where traditional medical practices coexist with biomedicine in a pluralistic healthcare system [13]. These cultural patterns of female genital cutting are often found in parts of the world where access to emergency obstetric care is poor and obstetric fistulas from obstructed labor are common [14].

For example, among the Hausa of northern Nigeria, there is a cultural belief in a gynecological condition called *gishiri*. *Gishiri* is the Hausa word for “salt,” and it refers to the common salt used in cooking, and to the chemical salts deposited at the bottoms of water jars as their contents evaporate. *Gishiri* (“salt”) plays a role in the traditional Hausa system of ethnomedicine, where it exists in balance with sweetness (*zaki*) and other humoral elements [15, 16]. The accumulation of “salt” in the vagina is thought to cause various ill-defined gynecological complaints, including dyspareunia, lack of interest in sexual activity, vaginal narrowing, and obstruction to birth during the second stage of labor. In such cases, the patient may be taken by family members to a barber (*wanzami*) or midwife (*unguzoma*), who performs a vaginal cutting procedure to eliminate the postulated condition. Usually, this involves incision of the anterior vagina and often results in accidental urethral injury. Case series of genito-urinary fistulas from northern Nigeria show *gishiri* cuts to account for between 1 and 18% of genito-urinary fistulas in this part of the world [5, 17–20]. In one analysis of the contribution of *gishiri* cutting to fistulas in northern Nigeria, the authors indicated that it has been performed for obstructed labor, to excise a perceived abnormal vaginal mass, and as a treatment for dyspareunia [19]. In one case, the patient exsanguinated as a result and died from hemorrhagic shock. In Tahzib’s series of 80 cases of fistula in adolescent girls aged 13 or younger, the procedure was performed for dyspareunia, amenorrhea, coital difficulties, abdominal pain, vulvar rashes, general ill health, fevers, and infertility [18]. He described these *gishiri* fistulas as “typically long and clean, mid-vaginal, with total or partial destruction of the urethra” [10], a description consistent with our findings in this series of cases.

Although the concept of *gishiri* does not travel across the Nigeria–Niger border, among the Hausa of southern Niger, a similar belief is called *gurya* (sometimes *angurya* or ‘*dan gurya*), a word generally translated as “cottonseed.” Here, the belief is that a girl may be born with a tiny “seed” within her genitalia that can expand or grow over time, eventually causing various problems affecting her physiology and disposition, such as lack of sexual desire or diminished vaginal capacity, which results in dyspareunia or even the inability to have vaginal intercourse at all. If *gurya* is diagnosed, the treatment, as with *gishiri*, is often a cutting procedure performed by a local barber.

Examination of the social circumstances of these 10 *gurya* cases reveals a very clear and disturbing pattern. For the most part, these patients with cutting-related fistulas were young women, all married as adolescents (range 12 to 16 years) who experienced repeated sexual trauma in the form of unwanted intercourse, with resulting marital disharmony, mental distress, and social discord. The early age of marriage is typical for this part of Africa, a social practice that often leads to pregnancy before pelvic growth is complete, predisposing many young brides to the development of obstructed labor and obstetric fistula formation (a common scenario in most series of obstetric fistulas [4–6]. However, none of the cases in this series was of obstetric origin and none appears to have been done as a rite of passage or a mandated cultural practice analogous to male circumcision, as is often true in many parts of Africa [11].

In contrast, these injuries appear to have resulted from cutting procedures that were intended to “open” the female genitalia so that the affected women would assume their culturally expected behaviors as willing (or at least tolerant) sexual partners for their husbands. In young adolescents who are married early, the vagina may not yet be sufficiently developed to allow atraumatic sexual intercourse, a fact that itself may lead to fistula formation [21]. In adolescent girls, especially those who are married without their consent, early unwanted intercourse may be both physically and emotionally traumatic, leading to anticipatory pain and guarding, which only serve to make subsequent sexual encounters more traumatic, starting a vicious psycho-sexual spiral from which they may never escape. A genital cutting procedure can only make this worse. Clinical interviews with many of the women in this series revealed the presence of co-existent anxiety and depression and in some cases post-traumatic stress disorder was suspected. Given their circumstances, this should not be surprising.

The underlying cultural assumption among the Hausa is that the proper role of women is to be wives. As wives, they should be submissive to their husband, seeking to please him both socially and sexually, and to provide him with many children as his “return on investment” from the marriage payments. Indeed, Hausa men regard women as “fields” to be “tilled” sexually, and the children that result are the “crop” or “profit” from such “tilling.” If the “field” does not wish to be “tilled” or finds the “tilling” to be unpleasant or painful, there must be something wrong with her—certainly psychosocially if not also biologically. There are also cases of women with *gurya* in whom the diagnosis was made after years of unsuccessful attempts at consensual vaginal penetration following marriage [22]. It was only after “therapeutic” cuttings produced genitourinary fistulas that caused their referral to hospitals that these women were diagnosed as having congenital abnormalities of the genitourinary tract. Indeed, *gurya* appears to be a diagnosis that is given to a wide range of female disorders in Nigerien Hausa society.

The fistula cases from genital cutting in this series are not the result of obstetrical trauma or a cultural rite of passage; rather, they are reflective of a society in which women are regarded primarily as sexual objects and incubators for children, rather than persons worthy in their own right, irrespective of their sexual attractiveness or personal characteristics. These fistulas are the result of a misguided therapeutic intent on the part of local traditional medical practitioners. All the injuries described in this paper were preventable, but any effective prevention strategy must pursue two complementary arms simultaneously: elevating the status of women in this part of the world, and at the same time providing them with access to competent, culturally acceptable obstetrical and gynecological care so that the need to access local cutting therapies of the type discussed here is no longer seen as necessary.
